# Baseline Sensitivity of *Echinochloa crus-gall* and *E. oryzicola* to Florpyrauxifen-Benzyl, a New Synthetic Auxin Herbicide, in Korea

**DOI:** 10.3389/fpls.2021.656642

**Published:** 2021-06-09

**Authors:** Soo-Hyun Lim, Harim Kim, Tae-Kyeong Noh, Ji-Soo Lim, Min-Jung Yook, Jin-Won Kim, Jee-Hwan Yi, Do-Soon Kim

**Affiliations:** ^1^Department of Plant Science, Department of Agriculture, Forestry and Bioresources, Research Institute of Agriculture and Life Sciences, College of Agriculture and Life Sciences, Seoul National University, Seoul, South Korea; ^2^Crop Protection Division, National Institute of Agricultural Sciences, Rural Development Administration, Jeonju, South Korea; ^3^Integrated Field Science, Corteva Agriscience, Indianapolis, IN, United States

**Keywords:** baseline sensitivity, *Echinochloa* species, florpyrauxifen-benzyl, herbicide resistance, Rinskor

## Abstract

*Echinochloa* species is one of the most problematic weed species due to its high competitiveness and increasing herbicide resistance. Florpyrauxifen-benzyl, a new auxin herbicide, was recently introduced for *Echinochloa* management; however, the potential risk for the development of herbicide resistance in *Echinochloa* species has not been well-investigated. Thus, this study was conducted to evaluate the baseline sensitivity of *Echinochloa* species to florpyrauxifen-benzyl to estimate the risk of future resistance development. A total of 70 and 71 accessions of *Echinochloa crus-galli* and *Echinochloa oryzicola* were collected from paddy fields in Korea, respectively. These two *Echinochloa* species were grown in plastic pots up to the 5-leaf stage, and treated with florpyrauxifen-benzyl at a range of doses from 2.2 g to 70.0 g a.i. ha^–1^. Nonlinear regression analyses revealed that GR_50_ values for *E. oryzicola* ranged from 4.54 g to 29.66 g a.i. ha^–1^, giving a baseline sensitivity index (BSI) of 6.53, while those for *E. crus-galli* ranged from 6.15 g to 16.06 g a.i. ha^–1^, giving a BSI of 2.61. Our findings suggest that *E. oryzicola* has a greater potential risk than *E. crus-galli* for the development of metabolism-based resistance to florpyrauxifen-benzyl.

## Introduction

The genus *Echinochloa* includes over 50 plant species, most of which are considered weeds in agricultural fields (Holm et al., 1977; Michael, 1983). These species are distributed in temperate to tropical regions and inhabit paddy and upland fields; therefore, they are important weeds for rice and upland crops (Barrett, 1983; Yabuno, 1983). Two *Echinochloa* species, *E. crus-galli* (barnyardgrass) and *E. oryzicola* (late watergrass), are predominantly found in Korean rice paddy fields and cause significant yield losses in rice cultivation (Gibson et al., 2002; Moon et al., 2010). *Echinochloa crus-galli* var. *crus-galli* inhabits paddy fields but mainly along the edge of paddy fields where the water depth is shallower. Similarly, *E. oryzicola* inhabits paddy fields but mainly inside paddy fields where the water depth is deeper. *Echinochloa crus-galli* var. *crus-galli* (*Echinochloa crus-galli* hereinafter) prefers wet soil rather than flooded water conditions, while *E. oryzicola* prefers flooded paddy fields due its high flooding adaptability (Kim, 1993; Nah et al., 2015). Their high competitiveness against rice (Moon et al., 2010, 2014) and dominance in paddy fields (Ha et al., 2014) have made *Echinochloa* species the most troublesome weed in paddy fields of rice cropping countries, including Korea.

Since the introduction of bensulfuron-methyl in 1987, acetolactate synthase (ALS) inhibitors have widely been used for paddy weed management due to their broad weed control spectrum and wide application window. Although azimsulfuron, bispyribac-sodium, imazosulfuron, and pyrazosulfuron-ethyl have herbicidal activity against *Echinochloa* species, flucetosulfuron and penoxsulam are the first ALS inhibitors claimed to have full activity against *Echinochloa* species. Acetyl CoA carboxylase (ACCase) inhibitors, particularly cyhalofop-butyl, have also been introduced to control *Echinochloa* species. However, the continuous and frequent use of ACCase and ALS inhibitors has resulted in herbicide-resistant *Echinochloa* species in Korea (Im et al., 2009; Kang et al., 2010; Kim, 2016; Song et al., 2017). Currently, herbicide-resistant *Echinochloa* species are distributed nationwide in Korean paddy fields (Lee et al., 2017; Lim et al., 2018). Herbicide resistance in *Echinochloa* species is not limited to Korea, but becomes a global issue in many rice cropping countries, such as the United States (Fischer et al., 2000; Norsworthy et al., 2014), Italy (Panozzo et al., 2013), Greece (Kaloumenos et al., 2013), Brazil (Matzenbacher et al., 2015), Japan (Iwakami et al., 2014), and China (Chen et al., 2016). Alternative herbicides with different modes of action are urgently required to manage the spread of herbicide-resistant *Echinochloa* species. Florpyrauxifen-benzyl (Rinskor^TM^ ^Active^, developed by Dow AgroSciences) is one of the alternatives. Florpyrauxifen-benzyl is a synthetic auxin herbicide of the arylpicolinate family (WSSA Group 4) that inhibits auxin action by binding to different sites than many existing auxin herbicides (Epp et al., 2016). Unlike general synthetic auxins, florpyrauxifen-benzyl has a broad herbicidal spectrum ranging from broadleaf weeds to grass and sedge weeds, with a particular activity against problematic weeds such as *Echinochloa* species, Palmer amaranth, and yellow nutsedge (Miller and Norsworthy, 2018). Due to its strong activity against *Echinochloa* species, it is also expected to control currently resistant *Echinochloa* species such as ALS and ACCase inhibitor-resistant *Echinochloa*, as well as quinclorac-resistant *Echinochloa* (Miller et al., 2018). However, the continuous and repeated use and sole reliance on this herbicide may eventually result in resistance to florpyrauxifen-benzyl. It is essential to estimate the potential risk of the development of resistance to a new herbicide. The response of each weed species to a specific herbicide differs between populations. The variation in genetic diversity and sensitivity is closely related to the possibility of the development of herbicide resistance in the species (Blows and Hoffmann, 2005; Moss, 2017). The baseline sensitivity test is mainly used to investigate the variation in sensitivity. As a definition of the EPPO standard PP1/213(2) (EPPO, 2003), baseline sensitivity data consider the variation in sensitivity among weed populations that have never been exposed to the herbicide or to related active substances with the same mode of action. The main objective of the baseline test is to investigate the natural variation in response to a specific chemical compound in a specific area or period. Nonetheless, a limited number of baseline sensitivity studies were conducted to evaluate the natural variations in response to new herbicides. The natural sensitivity variation of *Echinochloa* species was assessed for penoxsulam and other herbicides (Vidotto et al., 2004, 2007). Although a baseline sensitivity study requires substantial effort and resources, it is a useful tool to evaluate natural sensitivity variation in a particular weed species using populations from different regions and times and to provide us with an estimation of the potential risk for the development of herbicide resistance (Espeby et al., 2011).

Florpyrauxifen-benzyl is a new herbicide that was introduced to Korea in 2018. It is necessary to estimate the potential risk of resistance development, which is essential information to set up a strategy for sustainable use of the herbicide by delaying or minimizing resistance development. Therefore, this study was conducted to evaluate the natural variation in sensitivity to florpyrauxifen-benzyl among populations of *E. crus-galli* and *E. oryzicola* collected in Korean paddy fields before the introduction of florpyrauxifen-benzyl.

## Materials and Methods

### Plant Materials and Growing Conditions

**FIGURE 1:**
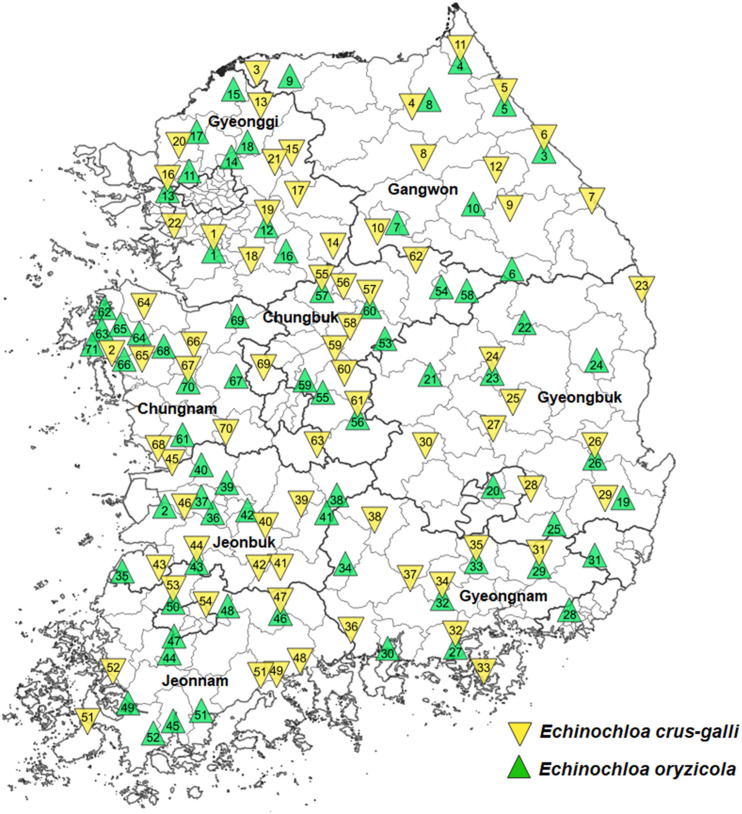
Collection sites of 70 *Echinochloa crus-galli* and 71 *Echinochloa oryzicola* accessions in Korea.

Among seeds collected from paddy fields in eight provinces of Korea between 2009 and 2016, a total of 70 accessions of *E. crus-galli* and 71 accessions of *E. oryzicola* were selected to represent 8–10 counties in each province (Figure 1). *Echinochloa crus-galli* and *E. oryzicola* were identified based on morphological traits (Lee et al., 2016). *Echinochloa crus-galli* accessions collected from Suwon and Seosan were previously confirmed to be susceptible and resistant to cyhalofop-butyl, respectively (Im et al., 2009), and *E. oryzicola* accessions collected from Suwon and Gimje were susceptible and resistant to penoxsulam, respectively (Kang et al., 2010; Song et al., 2017). Therefore, these accessions were used as reference accessions for this study. Collected accessions were stored in the cold chamber (4°C) to break dormancy until the seed was used. To harmonize seed germination, a priming treatment was made by keeping immersed seeds with distilled water in a cold chamber maintained at 4°C with no light for 48 h. The primed seeds of *Echinochloa* spp. were germinated in a 90 mm petri dish that was placed in a growth incubator under a 14-hour photoperiod with a 35/25°C day/night temperature for 72 h. Pregerminated seeds were transplanted into plastic pots (7 cm × 7 cm × 8 cm) filled with artificial paddy soil (Punong Co. Ltd., Gyeongju, South Korea) at a density of four plants per pot. All plants were placed in the glasshouse maintained at approximately 35/25°C day/night temperature until the final assessment at 30 days after treatment (DAT). Experiments were conducted in the glasshouse located at the Experimental Farm Station of Seoul National University, Suwon, South Korea.

### Dose-Response Study of *Echinochloa* Species to Florpyrauxifen-Benzyl (Whole-Plant Assay)

To investigate the baseline sensitivity of *Echinochloa* spp. to florpyrauxifen-benzyl, a dose-response study was conducted. Florpyrauxfen-benzyl (3.75% EC; Loyantε^TM^, KyungNong Co. Ltd., Seoul, South Korea) was applied to the foliage of *Echinochloa* accessions at the 5-leaf stage (plant height: 20–25 cm) at a range of doses from x1/16 to x2 the recommended dose of 35 g a.i. ha^–1^, and an untreated control was included. Herbicide application was completed using a compressor-pressurized belt-driven track sprayer (R&D Sprayers, United States) equipped with an 8002E flat-fan nozzle (Spraying Systems Company, United States) adjusted to deliver 500 L ha^–1^. After herbicide treatment, all the plants were placed in the glasshouse and arranged in a randomized block design with four replications. The shoot fresh weight after harvesting all the plants in a pot was measured at 30 days after treatment (DAT).

### Statistical Analysis

The shoot fresh weight data were examined using analysis of variance (ANOVA), and nonlinear regression was conducted to fit the data to the three-parameter log-logistic dose-response curve (Streibig, 1980) described as follows:

$$Y=\frac{Y_{o}}{1+{\left(\frac{x}{G\,R_{50}}\right)}^{B}}$$

where Y is the shoot fresh weight of *Echinochloa* spp., *x* is the herbicide dose, Y_o_ is the shoot fresh weight of untreated control, B is the slope of the curve, and the GR_50_ is the dose required for 50% shoot fresh weight reduction compared with untreated control. Using the parameter estimates, the GR_80_ values were also calculated. The baseline sensitivity index (BSI) was calculated by dividing the greatest GR_50_ and GR_80_ values (GR_50 max_ and GR_80 max_, respectively.) by the smallest values (GR_50 min_ and GR_80 min_).

The skewness and kurtosis were analyzed from the distribution of GR_50_ and GR_80_ values of all accessions (Pearson, 1905). The skewness is defined as follows:

$$\beta_{3}=\sum \frac{{\left(X_{i}-\bar{X}\right)}^{3}}{n\,s^{3}}$$

where n is the number of values, X_i_ is the i^th^ GR_50_ or GR_80_ value, $\bar{\text{X}}$ is the mean of GR_50_ or GR_80_ value, and s is the standard deviation. The kurtosis is defined as follows:

$$\beta_{4}=\sum \frac{{\left(X_{i}-\bar{X}\right)}^{4}}{n\,s^{4}}$$

where n is the number of values, X_i_ is the i^th^ GR_50_ or GR_80_ value, $\bar{\text{X}}$ is the mean of GR_50_ or GR_80_ value, and s is the standard deviation. All statistical analyses were conducted using Prism 7.04 (GraphPad Software, United States).

## Results

### Dose-Response of *Echinochloa* Species to Florpyrauxifen-Benzyl

**FIGURE 2:**
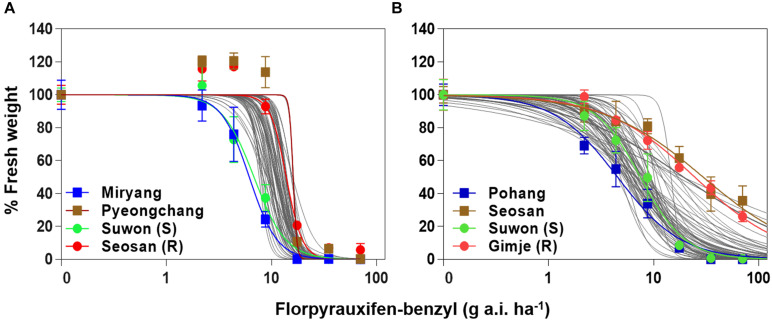
Dose-response curves in fresh weight (% control) of **(A)** *Echinochloa crus-galli* accessions and **(B)** *Echinochloa oryzicola* accessions measured at 30 days after florpyrauxifen-benzyl treatment.

Whole-plant assays with *Echinochloa* accessions and nonlinear regression analysis revealed dose responses of *E. crus-galli* and *E. oryzicola* to florpyrauxifen-benzyl, and showed sensitivity differences between *E. crus-galli* and *E. oryzicola* (Figure 2 and Supplementary Tables 1, 2). At the recommended dose (35 g a.i. ha^–1^) of florpyrauxifen-benzyl, 63 out of 70 tested *E. crus-galli* accessions (90%) were well-controlled with over 90% growth reduction in fresh weight compared to the untreated control, while 48 out of 71 tested *E. oryzicola* accessions (68%) were controlled (data not shown). At the double rate of the recommended dose, 8 *E. oryzicola* accessions (11%) were not well-controlled with less than 80% growth reduction, while all the tested *E. crus-galli* accessions were well-controlled with greater than 90% growth reduction. In *E. crus-galli*, Miryang accession from Gyeongnam province showed the greatest sensitivity with 6.15 g a.i. ha^–1^ of GR_50_, while Pyongchang accession from Gangwon province showed the lowest sensitivity with 16.06 g a.i. ha^–1^ of GR_50_ (Figure 2A and Supplementary Table 1), resulting in a 2.6-fold difference between them. Interestingly, two *E. crus-galli* reference accessions, Suwon and Seosan, previously confirmed susceptible and resistant, respectively, to both ACCase and ALS inhibitors (Im et al., 2009; Song et al., 2017), showed contrasting sensitivity to the herbicide. Suwon accession (susceptible reference) showed high sensitivity, similar to Miryang accession, with 6.81 g a.i. ha^–1^ of GR_50_, while Seosan accession (resistant reference) showed relatively low sensitivity with 13.83 g a.i. ha^–1^ of GR_50_ (Supplementary Table 1). In the case of *E. oryzicola*, Pohang accession from Gyeongbuk province showed the greatest sensitivity with 4.54 g a.i. ha^–1^ of GR_50_, while Seosan accession from Chungnam province showed the lowest sensitivity with 29.66 g a.i. ha^–1^ of GR_50_ (Figure 2B and Supplementary Table 2), resulting in a 6.5-fold difference between them. Similar to the case of *E. crus-galli*, two reference accessions, Suwon and Gimje previously confirmed susceptible and resistant, respectively, to both ACCase and ALS inhibitors (Zhang and Kim, 2016; Song et al., 2017), also showed contrasting sensitivity to the herbicide. Suwon accession (susceptible reference) showed greater sensitivity with 7.49 g a.i. ha^–1^ of GR_50_, than Gimje accession (resistant reference) with 24.62 g a.i. ha^–1^ of GR_50_ (Supplementary Table 2).

### Variation in Sensitivity of *Echinochloa* Species to Florpyrauxifen-Benzyl

**FIGURE 3:**
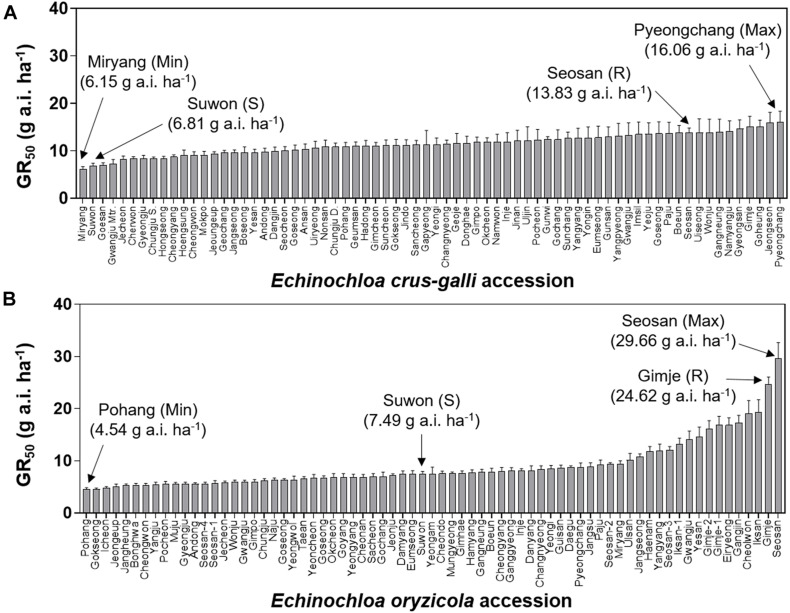
GR_50_ values of florpyrauxifen-benzyl for **(A)** *Echinochloa crus-galli* and **(B)** *Echinochloa oryzicola* accessions.

**FIGURE 4:**
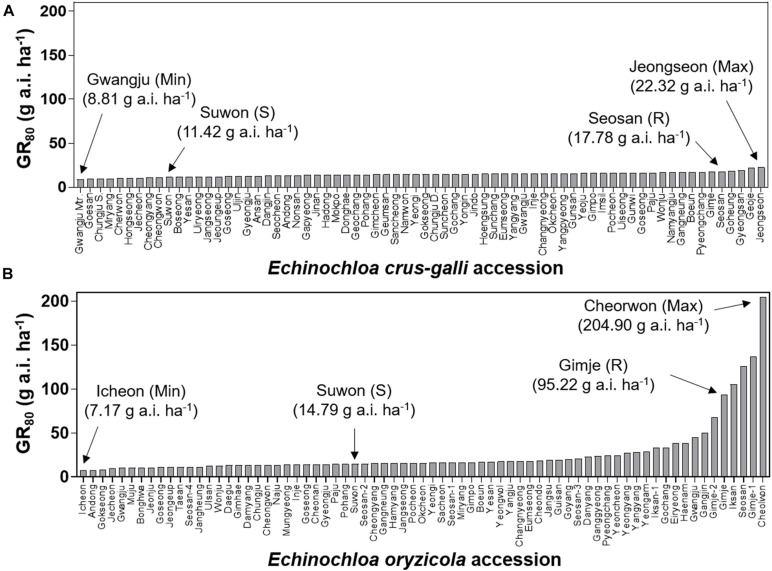
GR_80_ values of florpyrauxifen-benzyl for **(A)** *Echinochloa crus-galli* and **(B)** *Echinochloa oryzicola* accessions.

To compare variation in sensitivity of *Echinochloa* species to florpyrauxifen-benzyl, the GR_50_ values of *E. crus-galli* and *E. oryzicola* were arranged from the lowest to highest (Figure 3). In addition, the GR_80_ values were estimated and arranged because the GR_50_ value is derived from nonlinear regression to fit the log-logistic model and does not always represent the effective weed control dose, while the GR_80_ value is close to the effective weed control dose (Figure 4). The GR_50_ values of *E. crus-galli* ranged from 6.15 g to 16.06 g a.i. ha^–1^, while those of *E. oryzicola* ranged from 4.54 g to 29.66 g a.i. ha^–1^ (Figure 4), indicating that *E. oryzicola* has a wider variation in the GR_50_ value than *E. crus-galli*. In the case of GR_80_ values, variation in *E. crus-galli* ranging from 8.81 g to 22.32 g a.i. ha^–1^ was similar to that observed in GR_50_ values, while variation in *E. oryzicola* ranging from 7.17 g to 204.90 g a.i. ha^–1^ was greater than that in GR_50_ values, resulting in much larger variation in GR_80_ values in *E. oryzicola* than in *E. crus-galli* (Table 1).

**TABLE 1:** Summary of distribution analysis and baseline sensitivity in GR_50_ and GR_80_ values (g a.i. ha^–1^) of *Echinochloa* species in response to florpyrauxifen-benzyl.

Index	*Echinochloa crus-galli*		*Echinochloa oryzicola*	
		
	GR_50_	GR_80_	GR_50_	GR_80_
Kurtosis	–0.39	1.02	5.74	14.31
Skewness	–0.13	0.35	2.24	3.60
Mean	11.33	14.33	8.98	26.59
Median	11.35	14.56	7.49	15.65
Maximum (A)	16.06	22.32	29.66	204.90
Minimum (B)	6.15	8.81	4.54	7.17
Baseline sensitivity index (A/B)	2.61	2.53	6.53	28.58

When looking at individual accessions, the greatest GR_50_ value was 16.06 g a.i. ha^–1^ observed in the Pyeongchang accession of *E. crus-galli*, while the lowest value was 6.15 g a.i. ha^–1^ in the Miryang accession (Figure 3A). The highest GR_80_ value was 22.32 g a.i. ha^–1^ observed in the Jeongseon accession, and the lowest value was 8.81 g a.i. ha^–1^ in the Gwangju accession (Figure 4A). In the case of *E. oryzicola*, the greatest GR_50_ value was 29.66 g a.i. ha^–1^ observed in the Seosan accession, and the lowest value was 4.54 g a.i. ha^–1^ in the Pohang accession (Figure 3B). The highest GR_80_ value was 204.90 g a.i. ha^–1^ in the Cheorwon accession, and the lowest value was 7.17 g a.i. ha^–1^ in the Icheon accession (Figure 4B). These results suggest that *E. crus-galli* has no difference in variations between GR_50_ and GR_80_ values, while variation in *E. oryzicola* increased from 6.5 times in GR_50_ values to 28.6 times in GR_80_ values (Table 1). Additional dose response study of *E. oryzicola* accessions, Suwon, Gimje, and Cheorwon, at a range of florpyrauxifen-benzyl up to 140 g a.i. ha^–1^ also showed a similar sensitivity variation to florpyrauxifen-benzyl, GR_50_ ranging from 13.43 g to 85.59 g a.i. ha^–1^ and GR_80_ ranging from 18.15 g to 244.64 g a.i. ha^–1^ (Supplementary Table 3). Interestingly, GR_50_ and GR_80_ values were observed in resistant and susceptible resistant reference *E. crus-galli* and *E. oryzicola* accessions. In all cases, the GR_50_ and GR_80_ values of the resistant reference accession were always greater than those of the susceptible reference accession. All of the tested reference accessions were originally collected much earlier than the introduction of florpyrauxifen-benzyl and confirmed to be resistant to cyhalofop-butyl and penoxsulam.

### Baseline Sensitivity of *Echinochloa* Species to Florpyrauxifen-Benzyl

**FIGURE 5:**
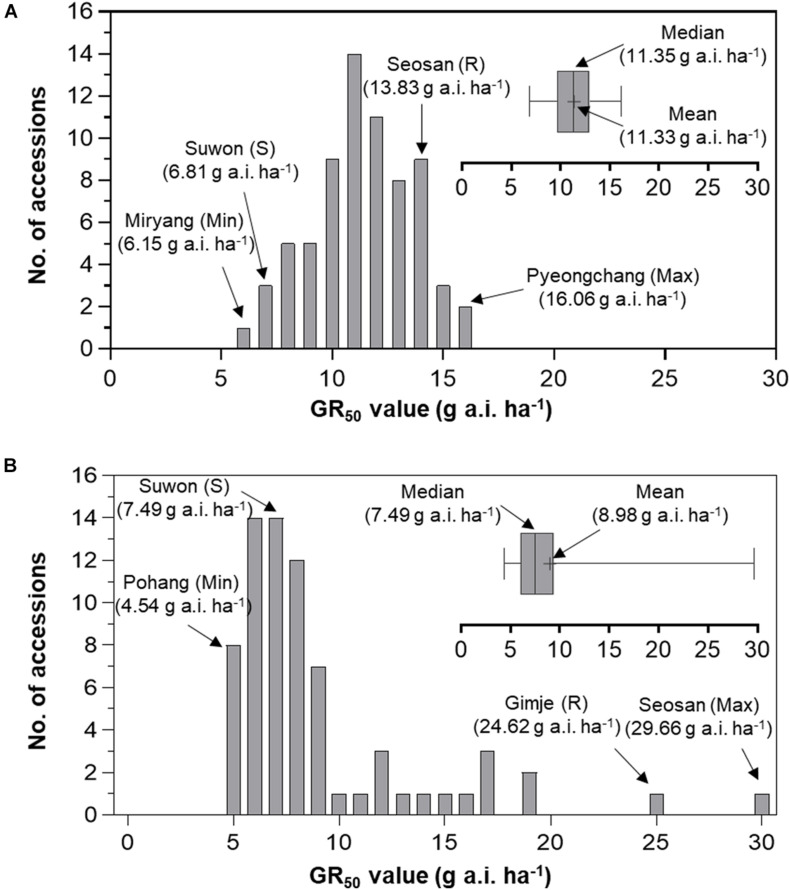
Distribution of GR_50_ values of florpyrauxifen-benzyl for **(A)** *Echinochloa crus-galli* and **(B)** *Echinochloa oryzicola* accessions.

**FIGURE 6:**
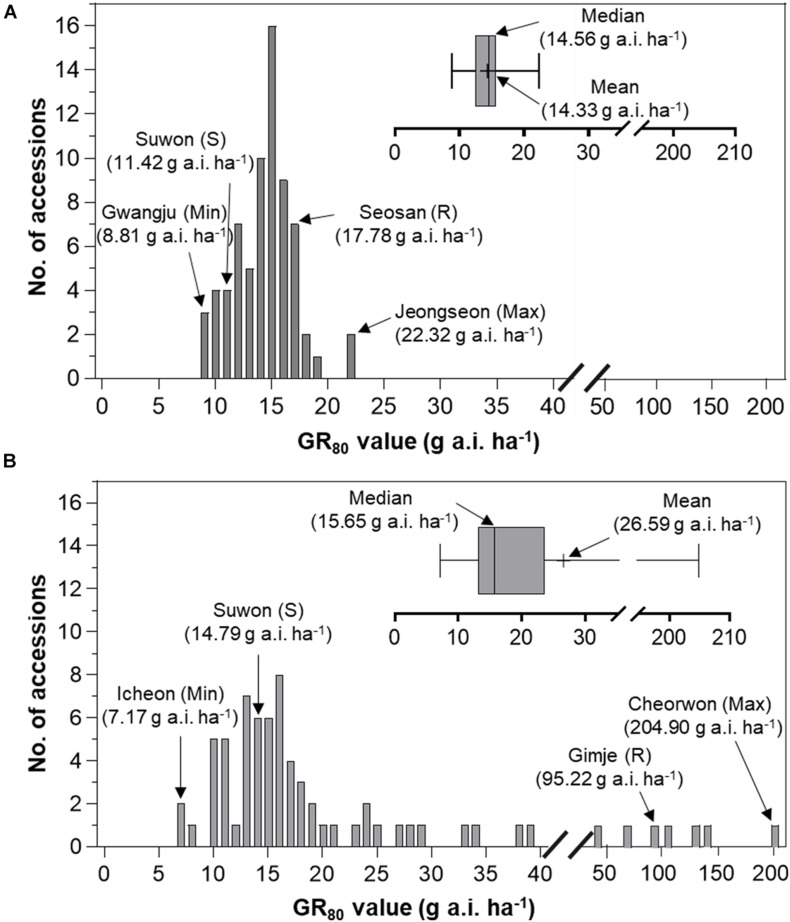
Distribution of GR_80_ values of florpyrauxifen-benzyl to **(A)** *Echinochloa crus-galli* and **(B)** *Echinochloa oryzicola* accessions.

Frequency distribution analysis of *E. crus-galli* and *E. oryzicola* GR_50_ and GR_80_ values was conducted to compare the baseline sensitivity of *Echinochloa* species to florpyrauxifen-benzyl (Figures 5, 6). The frequency distribution for the GR_50_ and GR_80_ values of *E. crus-galli* accessions followed a normal distribution with a narrow distribution range (Figures 5A, 6A), while those of *E. oryzicola* appeared to be a bimodal distribution with a much wider distribution range, almost 2 times the value and 8 times wider than *E. crus-galli* in GR_50_ and GR_80_ values, respectively (Figures 5B, 6B). The frequency distribution analysis based on normal distribution showed that *E. crus-galli* accessions were distributed following a normal distribution with mean and median values of 11.33 g and 11.35 g a.i. ha^–1^ for GR_50_, respectively, and values of 14.33 g and 14.56 g a.i. ha^–1^ for GR_80_, respectively (Table 1). In contrast, *E. oryzicola* did not follow a normal distribution considering its high kurtosis and skewness values for both GR_50_ and GR_80_ values. For example, the high *E. oryzicola* kurtosis values of 5.74 and 14.31 for GR_50_ and GR_80_ values, respectively, indicate a much wider range of distribution in GR_50_ and GR_80_ values of *E. oryzicola* accessions. Additionally, the high skewness values of 2.24 and 3.60 for GR_50_ and GR_80_ values, respectively, indicate right-skewed distribution and suggest that *E. oryzicola* has a wider sensitivity range than *E. crus-galli*.

To estimate the potential risk of development of florpyrauxifen-benzyl resistance in *Echinochloa* species, the BSI was calculated by dividing the greatest GR_50_ and GR_80_ values by the lowest GR_50_ and GR_80_ values, respectively. The BSI results for GR_50_ and GR_80_ values of *E. crus-galli* were 2.61 and 2.53, respectively, while those of *E. oryzicola* were 6.53 and 28.58, respectively (Table 1). In the case of BSI estimated based on the GR_50_ value, the difference between *E. crus-galli* and *E. oryzicola* was 2.50 times. As seen in Figure 5, both *E. crus-galli* and *E. oryzicola* accessions showed GR_50_ values less than 35 g a.i. ha^–1^, which is the recommended dose rate of florpyrauxifen-benzyl. However, when comparing the GR_80_ values, 9 accessions of *E. oryzicola* showed GR_80_ values greater than 35 g a.i. ha^–1^, suggesting that these accessions cannot be fully controlled by the recommended dose of florpyrauxifen-benzyl.

## Discussion

### Comparison and Implication of Baseline Sensitivity Between *Echinochloa* Species

The BSI of *E. oryzicola* was 2.5 times greater than that of *E. crus-galli* when estimated based on GR_50_ values, and it became 11.3 times greater when estimated based on GR_80_ values (Table 1). Therefore, *E. oryzicola* inhabiting paddy fields in Korea has a much greater potential of resistance development to florpyrauxifen-benzyl than *E. crus-galli*. The difference between BSI values estimated by GR_50_ and GR_80_ values suggests that the BSI should take into account both GR_50_ and GR_80_ values with greater importance in GR_80_ value, as it is more related to the recommended dose rate used in paddy fields. In addition, the frequency distribution of GR_50_ and GR_80_ values of *E. oryzicola* showed a right-skewed bimodal distribution, which is typical of creeping resistance (Gressel, 2009), with 9 accessions surviving above the recommended dose of florpyrauxifen-benzyl (Figure 6), while *E. crus-galli* showed a normal distribution with all of the *E. crus-galli* accessions effectively controlled by florpyrauxifen-benzyl. A similar right-skewed bimodal distribution was also observed in the GR_50_ frequency distribution of *E. crus-galli* and *E. oryzicola* in their dose responses to cyhalofop-butyl and penoxsulam, which confirmed that resistance to herbicides had already developed in *Echinochloa* species (Kim, 2016). Other studies also reported that *E. oryzicola* showed lower sensitivity to herbicides than *E. crus-galli* (Vidotto et al., 2007; Damalas et al., 2008; Song et al., 2017). Our findings, thus, suggest that *E. oryzicola* has a greater potential risk of resistance development for florpyrauxifen-benzyl due to its genetic difference; *E. oryzicola* is tetraploid (2n = 4X = 36), while *E. cru-galli* is hexaploid (2n = 6X = 54) (Gould et al., 1972).

The higher BSI and the right-skewed frequency distributions of GR_50_ and GR_80_ values observed in *E. oryzicola* might be due to the difference in the habitat and the selection pressure given. In general, *Echinochloa crus-galli* inhabits both dry and wet lands, while *E. oryzicola* inhabits flooded paddy fields, suggesting that the adaptability to submerged conditions is higher in *E. oryzicola* than *E. crus-galli* (Yabuno, 1960; Yamasue et al., 1989; Kim, 1993; Nah et al., 2015; Im, 2016). Since weed management for rice in Korea has been carried out by treating pre- or post-emergence herbicides directly to flooded paddy fields, it is likely that the entire population of *E. oryzicola* inhabiting flooded paddy fields has been well-exposed to herbicides for a long period of time, giving them a high selection pressure. However, many *E. crus-galli* plants inhabiting along the edge of paddy fields or banks of paddy fields have not been well-exposed to herbicides, giving them a low selection pressure. Yu and Powles (2014) suggested that NTSR may exhibit low sensitivity to herbicides with different modes of action, even first-time herbicides. Considering that the resistance mechanism of ACCase and ALS inhibitor-resistant *Echinochloa* species found in Korea was related to CYP450s-involved metabolism (Kim, 2016; Song et al., 2017), the low sensitivity (or insensitivity) to the new auxin herbicide florpyrauxifen-benzyl observed in some accessions of *E. oryzicola* in this study may also be related to CYP450s-involved metabolism. CYP450s-involved metabolism has been preselected by other herbicides, particularly ALS inhibitors, which have been used in rice fields in Korea for a long period of time. Other studies showed that florpyrauxifen-benzyl at the recommended standard dose could well control existing herbicide-resistant *E. crus-galli* populations in the Mekong delta of Vietnam (Duy et al., 2018) and in Arkansas, United States (Miller et al., 2018). However, our findings imply that *Echinochloa* populations with enhanced CYP450s-involved metabolism due to existing herbicides will become resistant to florpyrauxifen-benzyl eventually, depending on the use scenario. However, this should not exclude the involvement of reduced herbicide absorption and translocation.

### Sustainable Use of Florpyrauxifen-Benzyl for *Echinochloa* Management

Our study revealed that florpyrauxifen-benzyl could control *Echinochloa* species up to the 5-leaf stage and showed reasonably high activity against some ACCase and ALS inhibitor-resistant *Echinochloa* accessions, while was not effective against others. However, the baseline sensitivity study revealed that *E. oryzicola* has a relatively high BSI of 6.7 and many *E. oryzicola* accessions showed GR_80_ values greater than the recommended dose of florpyrauxifen-benzyl. Although the tested *Echinochloa* accessions have never been previously exposed to florpyrauxifen-benzyl, the high BSI suggests that *E. oryzicola* has a high potential risk of resistance development to florpyrauxifen-benzyl. If florpyrauxifen-benzyl is used solely and continuously for *Echinochloa* control, herbicide resistance will rapidly evolve due to the high selection pressure.

Although further studies are required to clearly elucidate the mechanism of this insensitivity, metabolism-based herbicide resistance might be a main mechanism for the insensitivity to florpyrauxifen-benzyl found in some *Echinochloa* accessions. Among the low sensitive or insensitive accessions tested in our study, some of them, including Seosan and Gimje, have already shown multiple resistances to ACCase and ALS inhibitors and cross-resistance to ALS inhibitors with different chemistries (Kim, 2016; Song et al., 2017). If florpyrauxifen-benzyl is intensively used with no rotation and/or mixtures with other herbicide modes of action, the risk of resistance development would increase. Sole reliance on a specific mode of action has resulted in the rapid development of herbicide resistance in *Echinochloa* species. Intensive use of penoxsulam in Gimje, South Korea, resulted in the rapid development of resistant *E. oryzicola* within 4 years of the commercial release of penoxsulam single a.i. product (Salchodaechup^TM^, Hankook Samgong, Korea) in 2004 (Kang et al., 2010; Park et al., 2010). Continuous use of limited herbicides such as propanil (PSII inhibitor) and bispyribac-sodium (ALS inhibitor) resulted in resistant *Echinochloa oryzicola* in the United States (Fischer et al., 2000). *Echinochloa colona* became resistant to propanil and fenoxaprop-P-ethyl due to the intensive use of these herbicides in Colombia and Costa Rica (Garro et al., 1991; Garita et al., 1995; Riches et al., 1996).

According to Busi et al. (2012), long-term selection pressure by a high dose of herbicide induces target site resistance, while that by a low dose of herbicide induces metabolic resistance. In our case, it is presumed that the insensitivity of *Echinochloa* species to florpyrauxifen-benzyl involves CYP450s-mediated metabolism although reduced herbicide absorption and translocation should also be another reason for the insensitivity. To avoid further resistance development and control resistant weeds, florpyrauxifen-benzyl needs to be used in a mixture or in rotation with other herbicides with different modes of action. Florpyrauxifen-benzyl applied in a mixture with acifluorfen, bentazon, carfentrazone, saflufencil, or propanil showed no antagonistic effect in Korean paddy fields, and shows good compatibility with other herbicides (Miller and Norsworthy, 2018). A recent study revealed that a florpyrauxifen-benzyl mixture with cyhalofop-butyl controlled paddy weeds well without residual toxicity to other crops or antagonistic effects (Sreedevi et al., 2020). Therefore, to maintain the sustainability of florpyrauxifen-benzyl in managing *Echinochloa* species in paddy fields, integrated weed management (IWM), including appropriate dosage (Busi et al., 2012), appropriate application timing, alternation of various modes of action (Evans et al., 2016), and cultural and physical methods (Bajwa, 2014; Weisberger et al., 2019), is required.

## Conclusion

The baseline sensitivity test for florpyrauxifen-benzyl against *E. crus-galli* and *E. oryzicola* demonstrated that *E. oryzicola* has a larger sensitivity variation and, thus, a greater potential for resistance development than *E. crus-galli*. The right-skewed frequency distributions of GR_50_ and GR_80_ values in *E. oryzicola* suggest that *E. oryzicola* can become resistant to florpyrauxifen-benzyl earlier than *E. crus-galli* if the herbicide is continuously and solely used for paddy weed management in Korea. Although the risk of resistance might be lower and the speed of resistance development slower in *E. crus-galli*, the continuous and sole use of the herbicide could also eventually lead to resistance. This potential risk of resistance development for florpyrauxifen-benzyl may not be limited to Korea because *Echinochloa* species have long been exposed to various herbicides in many rice cropping countries, particularly ACCase and ALS inhibitors, and ACCase and ALS inhibitor-resistant *Echinochloa* species are mostly involved with CYP450s-mediated metabolism. Although further studies are required to elucidate the mechanism of such baseline sensitivity differences between *E. oryzicola* and *E. crus-galli*, our results strongly support the diversity strategy of florpyrauxifen-benzyl, such as use in mixtures, in rotation with other herbicides with different modes of action, or in combination with diverse alternative nonchemical methods. The integrated use of florpyrauxifen-benzyl with other methods will be useful to maintain the sustainability of florpyrauxifen-benzyl as a rice herbicide.

## Data Availability Statement

The raw data supporting the conclusions of this article will be made available by the authors, without undue reservation, to any qualified researcher.

## Author Contributions

S-HL and HK conceived, conducted the experiments, and drafted and revised the manuscript. T-KN and J-SL helped to conduct the experiments. M-JY and J-WK helped to analyze the data and revised the manuscript. J-HY helped the funding acquisition and revised the manuscript. D-SK conceived, supervised the whole process of experiments, and wrote the manuscript. All authors critically reviewed the manuscript and approved the final version of the manuscript.

## Conflict of Interest

J-HY was employed by the company Corteva Agriscience. The authors declare that this study received funding from Corteva Agrisciecne. The funder had the following involvement in the study: study design and the decision to submit it for publication.

## Abbreviations

a.i.: active ingredient
GRxx: xx % of growth reduction
BSI: baseline sensitivity index
ALS: acetolactate synthase
ACCase: acetyl CoA carboxylase
EPPO: European and Mediterranean plant protection organization
DAT: days after treatment
ANOVA: analysis of variance
NTSR: non-target site resistance
CYP450s: cytochrome P450
IWM: integrated weed management.
