# Supplementary material for: Baseline Sensitivity of Echinochloa crus-gall and E. oryzicola to Florpyrauxifen-Benzyl, a New Synthetic Auxin Herbicide, in Korea
Source: Front Plant Sci. 2021 Jun 9;12:656642. doi: 10.3389/fpls.2021.656642 (PMC8221188; doi:10.3389/fpls.2021.656642)
Supplement: Supplementary file 1 [file Table_1.DOCX]

**Supplementary Table 1.** GR_50_ and GR_80_ values and statistical parameters in fresh weight of *Echinochloa crus-galli* accessions measured at 30 days after florpyrauxifen-benzyl treatment

| Province | Accession | Collection year | Accession code | No. | Statistical parameters | | | |
| --- | --- | --- | --- | --- | --- | --- | --- | --- |
|  |  |  |  |  | GR_50_ (g a.i. ha^-1^) | GR_80_ (g a.i. ha^-1^) | B | R^2^ |
| Reference | Suwon (S) | 2004 | SNU-E-01.005 | 1 | 6.81 (0.58) | 11.42 (1.13) | 2.85 (0.60) | 0.912 |
|  | Seosan (R) | 2009 | SNU-E-05.312 | 2 | 13.83 (0.99) | 17.78 (1.07) | 5.65 (1.46) | 0.940 |
| Gangwon | Cherwon | 2011 | SNU-E-08.081 | 3 | 8.29 (0.45) | 10.11 (1.14) | 6.98 (5.92) | 0.954 |
|  | Inje | 2011 | SNU-E-08.107 | 4 | 11.82 (2.02) | 15.32 (1.27) | 5.34 (2.72) | 0.761 |
|  | Yangyang | 2011 | SNU-E-08.114 | 5 | 12.70 (2.02) | 15.23 (1.17) | 7.64 (3.58) | 0.859 |
|  | Gangneung | 2011 | SNU-E-08.116 | 6 | 13.89 (2.75) | 16.77 (1.13) | 7.37 (5.85) | 0.797 |
|  | Donghae | 2011 | SNU-E-08.118 | 7 | 11.59 (1.44) | 14.12 (1.19) | 7.04 (2.82) | 0.888 |
|  | Hoengsung | 2016 | SNU-E-13.144 | 8 | 9.03 (1.11) | 15.10 (1.22) | 2.70 (0.86) | 0.825 |
|  | Jeongseon | 2016 | SNU-E-13.132 | 9 | 15.90 (2.18) | 22.32 (1.24) | 4.09 (2.45) | 0.733 |
|  | Wonju | 2016 | SNU-E-13.121 | 10 | 13.88 (2.77) | 16.59 (1.12) | 7.76 (6.29) | 0.815 |
|  | Goseong | 2016 | SNU-E-13.180 | 11 | 13.63 (2.49) | 16.47 (1.14) | 7.33 (4.94) | 0.771 |
|  | Pyeongchang | 2016 | SNU-E-13.134 | 12 | 16.06 (2.26) | 16.98 (1.33) | 4.93 (4.61) | 0.754 |
| Gyeonggi | Pocheon | 2011 | SNU-E-08.083 | 13 | 12.30 (2.18) | 16.01 (1.25) | 5.26 (2.68) | 0.730 |
|  | Yeoju | 2011 | SNU-E-08.202 | 14 | 13.52 (2.36) | 15.65 (1.12) | 9.47 (6.16) | 0.939 |
|  | Gapyeong | 2011 | SNU-E-08.317 | 15 | 11.35 (2.91) | 13.65 (1.48) | 7.52 (6.84) | 0.730 |
|  | Gimpo | 2011 | SNU-E-08.324 | 16 | 11.77 (1.10) | 14.22 (1.13) | 7.34 (2.09) | 0.952 |
|  | Yangpyeong | 2016 | SNU-E-13.080 | 17 | 13.06 (2.70) | 15.21 (1.17) | 9.10 (6.15) | 0.898 |
|  | Yongin | 2016 | SNU-E-13.041 | 18 | 12.73 (2.28) | 14.85 (1.17) | 9.01 (4.86) | 0.906 |
|  | Gwangju | 2016 | SNU-E-13.072 | 19 | 13.21 (2.69) | 15.27 (1.16) | 9.61 (6.62) | 0.913 |
|  | Paju | 2016 | SNU-E-13.101 | 20 | 13.67 (2.34) | 16.51 (1.13) | 7.35 (4.70) | 0.857 |
|  | Namyangju | 2016 | SNU-E-13.118 | 21 | 14.08 (2.20) | 16.59 (1.08) | 8.44 (5.84) | 0.929 |
|  | Ansan | 2016 | SNU-E-13.002 | 22 | 10.28 (1.17) | 12.61 (1.27) | 6.79 (4.40) | 0.907 |
| Gyeongbuk | Uljin | 2011 | SNU-E-08.121 | 23 | 12.11 (2.95) | 14.39 (1.33) | 8.04 (5.70) | 0.809 |
|  | Andong | 2011 | SNU-E-08.133 | 24 | 9.73 (0.79) | 13.16 (1.19) | 4.59 (1.94) | 0.879 |
|  | Uiseong | 2011 | SNU-E-08.136 | 25 | 13.85 (2.86) | 16.16 (1.11) | 8.97 (7.63) | 0.908 |
|  | Pohang | 2011 | SNU-E-08.140 | 26 | 10.89 (0.84) | 14.21 (1.14) | 5.22 (1.49) | 0.920 |
|  | Gunwi | 2011 | SNU-E-08.146 | 27 | 12.34 (0.62) | 16.36 (1.07) | 4.92 (0.71) | 0.965 |
|  | Gyeongsan | 2011 | SNU-E-08.152 | 28 | 14.63 (1.88) | 20.00 (1.17) | 4.43 (2.28) | 0.833 |
|  | Gyeongju | 2011 | SNU-E-08.154 | 29 | 8.30 (0.72) | 12.39 (1.16) | 3.46 (1.05) | 0.890 |
|  | Gimcheon | 2011 | SNU-E-08.180 | 30 | 11.07 (0.65) | 14.32 (1.10) | 5.39 (1.13) | 0.949 |
| Gyeongnam | Miryang | 2011 | SNU-E-08.158 | 31 | 6.15 (0.52) | 9.38 (1.14) | 3.28 (0.73) | 0.900 |
|  | Goseong | 2011 | SNU-E-08.165 | 32 | 10.11 (1.08) | 12.17 (1.26) | 7.46 (5.26) | 0.947 |
|  | Geoje | 2011 | SNU-E-08.168 | 33 | 11.58 (2.06) | 22.25 (1.33) | 2.12 (0.71) | 0.728 |
|  | Uiryeong | 2011 | SNU-E-08.170 | 34 | 10.56 (1.43) | 13.45 (1.28) | 5.73 (3.33) | 0.865 |
|  | Changnyeong | 2011 | SNU-E-08.175 | 35 | 11.44 (0.81) | 15.33 (1.11) | 4.73 (1.04) | 0.929 |
|  | Hadong | 2011 | SNU-E-08.275 | 36 | 10.99 (1.19) | 14.00 (1.20) | 5.73 (2.32) | 0.836 |
|  | Sancheong | 2011 | SNU-E-08.282 | 37 | 11.27 (0.91) | 14.39 (1.14) | 5.68 (1.56) | 0.919 |
|  | Geochang | 2011 | SNU-E-08.287 | 38 | 9.55 (0.53) | 14.13 (1.11) | 3.54 (0.72) | 0.946 |
| Jeonbuk | Jinan | 2011 | SNU-E-08.229 | 39 | 12.05 (2.26) | 13.90 (1.26) | 9.72 (5.43) | 0.910 |
|  | Imsil | 2011 | SNU-E-08.231 | 40 | 13.48 (2.58) | 15.73 (1.12) | 9.00 (6.36) | 0.933 |
|  | Namwon | 2011 | SNU-E-08.234 | 41 | 11.80 (1.67) | 14.53 (1.20) | 6.65 (2.83) | 0.876 |
|  | Sunchang | 2011 | SNU-E-08.237 | 42 | 12.64 (1.30) | 15.14 (1.11) | 7.67 (2.32) | 0.935 |
|  | Gochang | 2011 | SNU-E-08.263 | 43 | 12.34 (2.07) | 14.66 (1.21) | 8.03 (3.90) | 0.876 |
|  | Jeoungeup | 2011 | SNU-E-08.272 | 44 | 9.35 (0.45) | 11.99 (1.16) | 5.58 (2.55) | 0.951 |
|  | Gunsan | 2011 | SNU-E-08.309 | 45 | 12.93 (2.10) | 15.55 (1.16) | 7.51 (3.73) | 0.855 |
|  | Gimje | 2009 | SNU-E-06.022 | 46 | 15.05 (2.21) | 18.52 (1.10) | 6.68 (5.92) | 0.877 |
| Jeonnam | Gokseong | 2011 | SNU-E-08.238 | 47 | 11.09 (1.30) | 14.57 (1.21) | 5.08 (2.04) | 0.835 |
|  | Suncheon | 2011 | SNU-E-08.242 | 48 | 11.08 (1.22) | 14.61 (1.20) | 5.01 (1.89) | 0.841 |
|  | Goheung | 2011 | SNU-E-08.243 | 49 | 15.11 (1.30) | 18.56 (1.06) | 6.75 (3.64) | 0.942 |
|  | Boseong | 2011 | SNU-E-08.245 | 50 | 9.56 (0.92) | 11.97 (1.38) | 6.53 (6.08) | 0.899 |
|  | Jindo | 2011 | SNU-E-08.252 | 51 | 11.18 (1.15) | 14.95 (1.16) | 4.77 (1.54) | 0.860 |
|  | Mokpo | 2011 | SNU-E-08.254 | 52 | 9.07 (0.79) | 14.09 (1.16) | 3.15 (0.83) | 0.902 |
|  | Jangseong | 2011 | SNU-E-08.266 | 53 | 9.56 (0.63) | 11.88 (1.21) | 6.38 (3.99) | 0.942 |
|  | Gwangju Mtr. | 2011 | SNU-E-08.268 | 54 | 7.28 (0.89) | 8.81 (1.06) | 7.65 (4.81) | 0.940 |
| Chungbuk | Eumseong | 2011 | SNU-E-08.206 | 55 | 12.79 (2.38) | 15.19 (1.19) | 8.05 (4.49) | 0.844 |
|  | Chungju S. | 2011 | SNU-E-08.209 | 56 | 8.32 (0.34) | 10.23 (1.10) | 6.70 (3.96) | 0.973 |
|  | Chungju D. | 2011 | SNU-E-08.211 | 57 | 10.79 (0.84) | 14.59 (1.14) | 4.60 (1.27) | 0.917 |
|  | Goesan | 2011 | SNU-E-08.215 | 58 | 6.97 (0.47) | 9.84 (1.09) | 4.03 (0.89) | 0.940 |
|  | Cheongwon | 2011 | SNU-E-08.217 | 59 | 9.07 (0.74) | 11.99 (1.24) | 4.98 (3.28) | 0.858 |
|  | Boeun | 2011 | SNU-E-08.219 | 60 | 13.79 (1.51) | 16.95 (1.08) | 6.71 (2.84) | 0.929 |
|  | Okcheon | 2011 | SNU-E-08.221 | 61 | 11.77 (0.99) | 15.40 (1.13) | 5.15 (1.29) | 0.906 |
|  | Jecheon | 2011 | SNU-E-08.325 | 62 | 8.24 (0.64) | 10.65 (1.17) | 5.41 (3.81) | 0.881 |
| Chungnam | Geumsan | 2011 | SNU-E-08.224 | 63 | 10.96 (0.89) | 14.38 (1.14) | 5.10 (1.48) | 0.912 |
|  | Dangjin | 2011 | SNU-E-08.297 | 64 | 9.92 (0.84) | 12.73 (1.22) | 5.56 (2.92) | 0.900 |
|  | Hongseong | 2011 | SNU-E-08.302 | 65 | 8.33 (0.49) | 10.32 (1.16) | 6.48 (5.69) | 0.931 |
|  | Yesan | 2011 | SNU-E-08.304 | 66 | 9.65 (0.68) | 11.68 (1.22) | 7.25 (4.90) | 0.968 |
|  | Cheongyang | 2011 | SNU-E-08.306 | 67 | 8.72 (0.42) | 10.95 (1.22) | 6.08 (5.18) | 0.923 |
|  | Seocheon | 2011 | SNU-E-08.308 | 68 | 10.08 (0.81) | 12.75 (1.19) | 5.91 (2.80) | 0.929 |
|  | Yeongi | 2011 | SNU-E-08.311 | 69 | 11.35 (1.36) | 14.55 (1.20) | 5.59 (2.21) | 0.857 |
|  | Nonsan | 2009 | SNU-E-06.006 | 70 | 10.79 (1.46) | 13.24 (1.26) | 6.77 (3.88) | 0.897 |
