# Supplementary material for: Baseline Sensitivity of Echinochloa crus-gall and E. oryzicola to Florpyrauxifen-Benzyl, a New Synthetic Auxin Herbicide, in Korea
Source: Front Plant Sci. 2021 Jun 9;12:656642. doi: 10.3389/fpls.2021.656642 (PMC8221188; doi:10.3389/fpls.2021.656642)
Supplement: Supplementary file 2 [file Table_2.DOCX]

**Supplementary Table 2.** GR_50_ and GR_80_ values and statistical parameters in fresh weight of *Echinochloa oryzicola* accessions measured at 30 days after florpyrauxifen-benzyl treatment.

| Province | Accession | Collection  year | Accession code | No. | Statistical parameters | | | |
| --- | --- | --- | --- | --- | --- | --- | --- | --- |
|  |  |  |  |  | GR_50_ (g a.i. ha^-1^) | GR_80_ (g a.i. ha^-1^) | B | R^2^ |
| Reference | Suwon (S) | 2004 | SNU-E-01.004 | 1 | 7.49 (0.33) | 14.79 (1.07) | 2.02 (0.16) | 0.978 |
|  | Gimje (R) | 2009 | SNU-E-01.009 | 2 | 24.62 (1.84) | 95.22 (1.16) | 1.04 (0.09) | 0.940 |
| Gangwon | Gangneung | 2011 | SNU-E-08.115 | 3 | 7.77 (0.57) | 15.45 (1.13) | 2.02 (0.27) | 0.940 |
|  | Goseong | 2011 | SNU-E-08.110 | 4 | 6.26 (0.25) | 10.72 (1.07) | 2.58 (0.24) | 0.975 |
|  | Yangyang | 2011 | SNU-E-08.113 | 5 | 11.76 (1.10) | 28.39 (1.18) | 1.58 (0.21) | 0.887 |
|  | Yeongwol | 2011 | SNU-E-08.099 | 6 | 6.22 (0.62) | 18.15 (1.19) | 1.29 (0.17) | 0.897 |
|  | Wonju | 2011 | SNU-E-08.092 | 7 | 5.91 (0.35) | 12.70 (1.10) | 1.82 (0.18) | 0.962 |
|  | Inje | 2011 | SNU-E-08.108 | 8 | 8.11 (0.40) | 13.73 (1.08) | 2.66 (0.31) | 0.969 |
|  | Cheorwon | 2011 | SNU-E-08.080 | 9 | 19.03 (2.48) | 204.90 (1.46) | 0.47 (0.05) | 0.911 |
|  | Pyeongchang | 2011 | SNU-E-08.103 | 10 | 8.71 (0.79) | 23.96 (1.16) | 1.35 (0.16) | 0.920 |
| Gyeonggi | Goyang | 2011 | SNU-E-08.069 | 11 | 6.83 (0.71) | 19.95 (1.18) | 1.21 (0.15) | 0.903 |
|  | Gwangju | 2011 | SNU-E-08.192 | 12 | 14.16 (1.55) | 45.26 (1.21) | 1.16 (0.15) | 0.874 |
|  | Gimpo | 2011 | SNU-E-08.323 | 13 | 5.99 (0.42) | 16.38 (1.12) | 1.31 (0.12) | 0.956 |
|  | Yangju | 2011 | SNU-E-08.086 | 14 | 5.41 (0.48) | 17.46 (1.15) | 1.10 (0.12) | 0.939 |
|  | Yeoncheon | 2011 | SNU-E-08.079 | 15 | 6.68 (0.69) | 24.61 (1.19) | 0.99 (0.11) | 0.920 |
|  | Icheon | 2011 | SNU-E-08.196 | 16 | 4.83 (0.24) | 7.17 (1.09) | 3.51 (0.59) | 0.958 |
|  | Paju | 2011 | SNU-E-08.074 | 17 | 9.29 (0.88) | 14.48 (1.17) | 3.12 (0.88) | 0.882 |
|  | Pocheon | 2011 | SNU-E-08.085 | 18 | 5.52 (0.54) | 15.61 (1.17) | 1.28 (0.16) | 0.924 |
| Gyeongbuk | Gyeongju | 2011 | SNU-E-08.153 | 19 | 5.54 (0.35) | 14.34 (1.10) | 1.40 (0.12) | 0.965 |
|  | Daegu | 2011 | SNU-E-08.147 | 20 | 8.72 (0.31) | 13.16 (1.06) | 3.37 (0.41) | 0.978 |
|  | Mungyeong | 2011 | SNU-E-08.186 | 21 | 7.56 (0.30) | 13.69 (1.07) | 2.33 (0.20) | 0.980 |
|  | Bonghwa | 2011 | SNU-E-08.128 | 22 | 5.30 (0.45) | 10.45 (1.11) | 2.00 (0.25) | 0.950 |
|  | Andong | 2011 | SNU-E-08.134 | 23 | 5.55 (0.22) | 7.48 (1.07) | 4.66 (0.61) | 0.972 |
|  | Yeongyang | 2011 | SNU-E-08.125 | 24 | 6.84 (0.58) | 27.38 (1.16) | 0.91 (0.08) | 0.945 |
|  | Cheongdo | 2011 | SNU-E-08.149 | 25 | 7.54 (0.48) | 18.25 (1.11) | 1.54 (0.14) | 0.960 |
|  | Pohang | 2011 | SNU-E-08.139 | 26 | 4.54 (0.30) | 14.51 (1.31) | 1.43 (0.13) | 0.967 |
| Gyeongnam | Goseong | 2011 | SNU-E-08.166 | 27 | 6.76 (0.37) | 13.89 (1.09) | 1.91 (0.18) | 0.968 |
|  | Gimhae | 2011 | SNU-E-08.162 | 28 | 7.59 (0.48) | 13.17 (1.10) | 2.51 (0.35) | 0.946 |
|  | Miryang | 2011 | SNU-E-08.157 | 29 | 9.39 (0.60) | 16.31 (1.11) | 2.51 (0.36) | 0.948 |
|  | Sacheon | 2011 | SNU-E-08.278 | 30 | 6.98 (0.58) | 16.17 (1.14) | 1.63 (0.20) | 0.934 |
|  | Ulsan | 2011 | SNU-E-08.156 | 31 | 10.13 (1.31) | 12.28 (1.33) | 7.22 (6.00) | 0.890 |
|  | Eiryeong | 2011 | SNU-E-08.171 | 32 | 16.84 (1.39) | 37.98 (1.14) | 1.70 (0.22) | 0.913 |
|  | Changnyeong | 2011 | SNU-E-08.174 | 33 | 8.39 (0.65) | 17.66 (1.13) | 1.86 (0.24) | 0.939 |
|  | Hamyang | 2011 | SNU-E-08.289 | 34 | 7.78 (0.50) | 15.45 (1.11) | 2.01 (0.50) | 0.954 |
| Jeonbuk | Gochang | 2011 | SNU-E-08.264 | 35 | 6.98 (0.86) | 33.53 (1.24) | 0.78 (0.09) | 0.900 |
|  | Gimje-1 | 2009 | SNU-E-06.030 | 36 | 16.83 (1.68) | 136.5 (1.30) | 0.56 (0.05) | 0.932 |
|  | Gimje-2 | 2009 | SNU-E-06.032 | 37 | 16.15 (1.53) | 67.49 (1.20) | 0.93 (0.09) | 0.914 |
|  | Muju | 2011 | SNU-E-08.226 | 38 | 5.53 (0.32) | 10.26 (1.10) | 2.22 (0.26) | 0.959 |
|  | Iksan | 2009 | SNU-E-06.015 | 39 | 19.25 (2.45) | 105.30 (1.32) | 0.76 (0.09) | 0.866 |
|  | Iksan-1 | 2009 | SNU-E-06.012 | 40 | 13.22 (1.14) | 33.04 (1.15) | 1.50 (0.18) | 0.920 |
|  | Jangsu | 2011 | SNU-E-08.227 | 41 | 8.83 (0.80) | 18.84 (1.16) | 1.82 (0.27) | 0.911 |
|  | Jeonju | 2011 | SNU-E-08.230 | 42 | 7.21 (0.30) | 10.46 (1.06) | 3.72 (0.49) | 0.971 |
|  | Jeongeup | 2011 | SNU-E-08.271 | 43 | 5.10 (0.43) | 10.73 (1.14) | 1.82 (0.26) | 0.927 |
| Jeonnam | Naju | 2009 | SNU-E-01.010 | 44 | 6.28 (0.45) | 13.47 (1.12) | 1.80 (0.21) | 0.948 |
|  | Gangjin | 2011 | SNU-E-08.249 | 45 | 17.28 (1.44) | 50.41 (1.15) | 1.28 (0.13) | 0.921 |
|  | Gokseong | 2011 | SNU-E-08.239 | 46 | 4.55 (0.22) | 7.85 (1.08) | 2.50 (0.28) | 0.967 |
|  | Gwangju Mtr. | 2011 | SNU-E-08.267 | 47 | 5.93 (0.34) | 9.92 (1.10) | 2.69 (0.36) | 0.955 |
|  | Damyang | 2011 | SNU-E-08.269 | 48 | 7.44 (0.61) | 13.20 (1.14) | 2.41 (0.42) | 0.918 |
|  | Yeongam | 2011 | SNU-E-08.257 | 49 | 7.49 (1.30) | 28.92 (1.34) | 0.98 (0.18) | 0.808 |
|  | Jangseong | 2011 | SNU-E-08.265 | 50 | 10.81 (0.54) | 15.59 (1.08) | 3.78 (0.58) | 0.953 |
|  | Jangheung | 2011 | SNU-E-08.295 | 51 | 5.28 (0.28) | 11.35 (1.09) | 1.77 (0.16) | 0.970 |
|  | Haenam | 2011 | SNU-E-08.250 | 52 | 11.82 (0.92) | 38.54 (1.14) | 1.14 (0.10) | 0.940 |
| Chungbuk | Goesan | 2011 | SNU-E-08.214 | 53 | 8.61 (0.58) | 19.11 (1.11) | 1.72 (0.18) | 0.954 |
|  | Danyang | 2011 | SNU-E-08.097 | 54 | 8.13 (0.90) | 22.76 (1.20) | 1.31 (0.18) | 0.891 |
|  | Boeun | 2011 | SNU-E-08.218 | 55 | 7.81 (0.77) | 16.76 (1.17) | 1.80 (0.29) | 0.905 |
|  | Okcheon | 2011 | SNU-E-08.220 | 56 | 6.80 (0.76) | 15.65 (1.20) | 1.65 (0.28) | 0.888 |
|  | Eumseong | 2011 | SNU-E-08.207 | 57 | 7.46 (0.66) | 17.75 (1.15) | 1.57 (0.21) | 0.926 |
|  | Jecheon | 2011 | SNU-E-08.096 | 58 | 5.84 (0.26) | 9.76 (1.07) | 2.69 (0.28) | 0.973 |
|  | Cheongwon | 2011 | SNU-E-08.216 | 59 | 5.30 (0.36) | 13.33 (1.11) | 1.45 (0.14) | 0.958 |
|  | Chungju | 2011 | SNU-E-08.210 | 60 | 6.23 (0.36) | 13.27 (1.10) | 1.80 (0.18) | 0.962 |
| Chungnam | Ganggyeong | 2009 | SNU-E-06.008 | 61 | 8.07 (0.64) | 23.57 (1.14) | 1.26 (0.12) | 0.944 |
|  | Seosan | 2008 | SNU-E-05.023 | 62 | 29.66 (3.04) | 125.8 (1.24) | 0.93 (0.10) | 0.889 |
|  | Seosan-1 | 2008 | SNU-E-05.144 | 63 | 5.71 (0.48) | 16.29 (1.14) | 1.26 (0.14) | 0.942 |
|  | Seosan-2 | 2008 | SNU-E-05.173 | 64 | 9.37 (0.26) | 14.84 (1.05) | 3.02 (0.24) | 0.989 |
|  | Seosan-3 | 2008 | SNU-E-05.175 | 65 | 12.10 (0.64) | 20.87 (1.09) | 2.54 (0.30) | 0.962 |
|  | Seosan-4 | 2008 | SNU-E-05.182 | 66 | 5.61 (0.28) | 11.14 (1.08) | 1.99 (0.19) | 0.970 |
|  | Yeongi | 2011 | SNU-E-08.310 | 67 | 8.48 (0.64) | 15.94 (1.13) | 2.19 (0.32) | 0.935 |
|  | Yesan | 2011 | SNU-E-08.303 | 68 | 14.63 (1.81) | 15.92 (1.03) | 9.54 (6.54) | 0.980 |
|  | Cheonan | 2011 | SNU-E-08.090 | 69 | 6.87 (0.52) | 14.31 (1.13) | 1.87 (0.24) | 0.940 |
|  | Cheongyang | 2011 | SNU-E-08.305 | 70 | 8.03 (0.68) | 15.26 (1.14) | 2.15 (0.35) | 0.918 |
|  | Taean | 2011 | SNU-E-08.299 | 71 | 6.54 (0.45) | 11.06 (1.11) | 2.63 (0.41) | 0.936 |
