# Supplementary material for: Baseline Sensitivity of Echinochloa crus-gall and E. oryzicola to Florpyrauxifen-Benzyl, a New Synthetic Auxin Herbicide, in Korea
Source: Front Plant Sci. 2021 Jun 9;12:656642. doi: 10.3389/fpls.2021.656642 (PMC8221188; doi:10.3389/fpls.2021.656642)
Supplement: Supplementary file 3 [file Table_3.docx]

**Supplementary Table 3.** Summary of GR_50_ and GR_80_ values (g a.i. ha^-1^) of selected *E. oryzicola* in response to florpyrauxifen-benzyl

| Collection site | GR_50_ | GR_80_ | B | R^2^ |
| --- | --- | --- | --- | --- |
| Suwon | 13.43 | 18.15 | 4.60 (1.29)^*^ | 0.824 |
| Gimje | 32.49 | 55.89 | 2.56 (0.44) | 0.902 |
| Cheorwon | 85.59 | 244.64 | 1.32 (0.28) | 0.765 |

^*^ Standard error
